# Effects of prenatal stress on reproductive function of male offspring through the KISS1 system

**DOI:** 10.1530/EC-24-0027

**Published:** 2024-09-13

**Authors:** Jian Gong, Yinjuan Lv, Yuhao Meng, Weiheng Zhang, Xiaocui Jiang, Min Xiao

**Affiliations:** 1 School of Basic Medicine, Hubei University of Chinese Medicine, Wuhan, China; 2 Hubei University of Chinese Medicine, Wuhan, China; 3 Laboratory Animal Center, Hubei University of Chinese Medicine, Wuhan, China

**Keywords:** HPG, KISS1, KISS1-R, prenatal stress, reproductive function

## Abstract

Prenatal stress can lead to the programming of the neuroendocrine system in male offspring, disrupting the hypothalamic testicular axis and adversely affecting the reproductive health of male offspring. This study aimed to determine the long-term effects of prenatal stress on the KISS1 system in male offspring and the effects on reproductive function in male offspring. Sixteen pregnant females were divided into a prenatal control group (PC, *n* = 8) and a prenatal stress group (PS, *n* = 8). The PS group was modeled with chronic unpredictable mild stress (CUMS) from day 1 of gestation to full-term delivery. Differences between the two groups in various maternal parameters, including glucocorticoid secretion, litter size, and the effects of male offspring birth weight, the KISS1 system, and reproductive function, were determined. Male offspring of PS dams had lower birth weights compared to prenatal controls.KISS1 gene expression is reduced at birth and in adult PS offspring, and its receptor KISS1-R protein is similarly reduced in PS offspring at birth and adulthood. In adulthood, PS male offspring show significantly reduced sex hormone production, altered testicular morphology, reduced maturation of their supporting cells, and decreased expression of connexin 43 (CX43), leading to an altered sperm microenvironment and reduced sperm quality. In conclusion, prenatal stress leads to adverse changes in the KISS1 system in male offspring and decreased reproductive function.

## Introduction

Prenatal stress (PS) is the body's response when various internal and external factors stimulate a woman during pregnancy (1). Studies have shown that when women are exposed to stressful events during pregnancy, they can disrupt their offspring's reproductive development and function and cause genetic defects (2). In humans, female exposure to stressful life events (SLE) during early gestation (18 weeks of gestation) is associated with reduced reproductive function in male offspring, such as reduced total sperm count and sperm motility, as well as reduced serum testosterone concentrations (3). In one study, prenatal stress in the form of bereavement increased the risk of reproductive disorders due to congenital malformations of the male offspring's reproductive organs (4). In animal models, prenatal stress led to apoptosis of testicular cells and reduced sperm quality and fertility in offspring rats (5). Maternal stress in sheep during late pregnancy affects sperm quality in the offspring during early puberty (6). There is growing evidence that stressors during pregnancy can impair male reproductive function in offspring. However, the neuroendocrine mechanisms are unclear.

Glucocorticoids (GCs) are major effectors of the stress response and play an essential role in regulating the stress response as an end product of the hypothalamic-pituitary-adrenal axis (HPA) (7). During pregnancy, antenatal stress induces increased maternal glucocorticoid levels (8), activates the GC-C/EBPα-Egr1 signaling pathway, decreases male placental 11β-hydroxysteroid dehydrogenase 2 (11β-HSD2) expression (9) and increases placental permeability (10), leading to antenatal intrauterine glucocorticoid exposure in the fetus. When exposed prenatally, glucocorticoids can disrupt the development and movement and alter the synaptic input of early offspring GnRH neurons (11, 12, 13), affecting the sexual development of the offspring and interfering with the differentiation of the hypothalamic-pituitary-gonadal axis (HPG) (14), which is known as prenatal programming of neuroendocrine reproductive function (15). The HPG axis is controlled by gonadotropin-releasing hormone (GnRH), which binds to receptors on the pituitary gonadotropic cord and stimulates the synthesis and secretion of follicle-stimulating hormone (FSH) (16). FSH binds to testicular support cells (Sertoli cells), which build the microenvironment for spermatogenesis and, together with testosterone, regulate the maturation of sperm cells (17). Within the reproductive neuroendocrine system, the Kiss1 system, located in the arcuate nucleus of the hypothalamus, is thought to play an essential role in controlling reproductive function during puberty and adulthood. This system includes kisspeptin and its receptor GPR54 (KISS1R) (18). Kisspeptins, a neuropeptide encoded by the Kiss1 gene, act as crucial central regulators of GnRH release to facilitate GnRH secretion and play an essential role in activating the reproductive axis during puberty and the regulation of gonadal function in adulthood (19). Alterations in the KISS1 system are closely related to reproductive function. They can lead to clinical conditions such as idiopathic hypogonadism (iHH), central precocious puberty (CPP), and male infertility when there is a mutation in the KISS1 gene or a disruption of the Kiss1 system (20, 21). However, it is not known whether prenatal stress affects the KISS1 system in male offspring.

This study investigated the effects of prenatal stress on the reproductive function of male offspring. The expression levels of kisspeptin and its receptor GPR54 were also assessed at birth and adulthood to determine prenatal stress’s effects on the KISS1 system in male offspring.

## Materials and methods

### Instruments and reagents

Rat glucocorticoid (GC), rat gonadotropin-releasing hormone (GnRH), rat follicle-stimulating hormone (FSH), rat anti-müllerian hormone (AMH) ELISA kits (Shanghai Jianglai Bio-technology Co., Ltd., Item No. JL27073, JL12201, JL13251, JL12462). β-actin protein antibody, horseradish peroxidase (HRP) enzyme-labeled goat anti-rabbit immunoglobulin G (IgG), ultrasensitive electrogenerated chemiluminescence (ECL) chemiluminescence kit, animal testis tissue fixation solution, hematoxylin-eosin (Wuhan Sevier Biotech Ltd., Item No. GB11002, GB2323). Labeled goat anti-rabbit immunoglobulin G (IgG), ultrasensitive electrogenerated chemiluminescence (ECL) kit, animal testis tissue fixative, hematoxylin-eosin (Wuhan Xavier Biotechnology Co., Ltd., Article No. GB11002, GB23303, G2020, G1121, G1076). antibody to KISS1-R protein, antibody to glucocorticoid receptor (GR) protein, antibody to Bax protein, antibody to BCL-2 protein, antibody to Caspase-3 protein, antibody to Bax protein, antibody to BCL-2 protein, antibody to Caspase-3 protein, antibody to Caspase-3 protein, antibody to Bax protein, antibody to BCL-2 protein, Caspase-3 Protein Antibody, FSH-R Protein Antibody, and CX43 Protein Antibody (Abclonal, Item No. A2967, A19583, A19684, A20777, A0214, A3172, A11752).

### Experimental animals

In this study, 16 SPF-grade 8-week-old Sprague-Dawley (SD) female rats with body mass (250 ± 20) g were used, purchased from the Animal Center of Three Gorges University, with license No. SCXK (E) 2017-0012, and experiments were carried out in the Animal Experiment Center of Hubei University of Traditional Chinese Medicine, with license No. SCXK (E) 2017-0067. Animals are maintained at a constant temperature (20–24°C) on a constant 12-hour light/12-hour darkness cycle (lighting from 08:00 h to 20:00 h) with free access to a standardized diet and water supply. The rats were housed for one week to acclimate to the environment before establishing the model.

### Animal grouping

Sixteen SD female rats were randomly divided into two groups using the completely randomized numerical grouping method: prenatal control (PC), and prenatal stress (PS), with eight rats in each group, housed at 1 per cage.

### Mating

Sixteen sexually experienced male rats were housed 1:1 in cages for natural male/female mating. Female rats examined vaginal plugs and vaginal smears to determine if the pregnancy was successful, and when pregnancy was confirmed, this day was considered day 0 of pregnancy. On day 1 of pregnancy, female rats in the PS group began to establish an animal model of prenatal stress.

### Chronic unpredictable moderate stress (CUMS) to establish an animal model of prenatal stress

**Figure 1:**
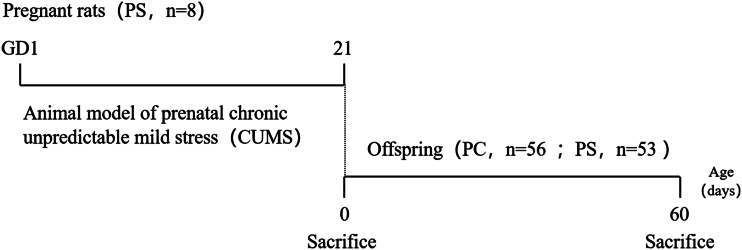
Study protocol. Female rats were judged to be successfully pregnant by examining vaginal plugs and vaginal smears, and after pregnancy was confirmed, the day was considered as day 0 of pregnancy, and when pregnancy was day 1, female rats in the PS group were initiated with chronic unpredictable mild stress (CUMS) in order to establish an animal model of prenatal stress, and the modeling cycle lasted a total of 21 days. The day of birth of the pups was considered as day 0 (PND 0), and the pups were examined for body weight, sex ratio, and litter size, where some male offspring (*n* = 7–8) were randomized for brain sampling at PND 0. Other male offspring from PC and PS groups received normal feeding until day 60 when males were executed as adults. PC group, prenatal control group; PS group, prenatal stress group.

The CUMS method established a prenatal stress model in the literature (22). The specific modeling details are as follows: the modeling period is 21d. The nine stimulation methods are arranged randomly to 21d. In principle, the same stress pattern does not occur within 7d. Among the specific methods are: i) food deprivation for 24 h; ii) water deprivation for 24 h; iii) crowded environment (24 h, cage tilted at 30 degrees); iv) humid environment with 60–70% humidity for 24 h; v) hot water swimming at 31°C for 1 h; vi) shaking for 30 min at a time; vii) behavioral restraint for 30 min; viii) heat stress at 42°C for 5 min; ix) tail clamping (1 cm from the tip of the tail for 1 min). Except for i, ii, iii, and iv, (24 h to implement the stress method), the rest of the day will occur between 10:00 h and 12:00 h, The 9 molding methods continued to mold for 21 days with no days off, just a different molding method each day. After the stress was complete, the rats were returned to their original breeding environment. The research program is shown in Fig. 1.

### Assessment of prenatal stress model

On day 21 of pregnancy, females were subjected to sucrose preference and open field experiments to assess molding success. Among them, the PC and PS groups randomly picked three female rats each for serum and male placenta sampling.

### Open-field test

This experiment was carried out in conjunction with the sucrose preference experiment. The open field apparatus is made of opaque material and has a 40cm × 40cm square at the bottom, divided equally into nine equilateral squares. How to do it: after 5 min of acclimatization in a quiet environment, the female is placed in the central square, and her horizontal locomotion score, vertical locomotion score, and the number of times she enters the central area during the 5 min period are recorded. (After each rat, wipe the bottom of the box thoroughly with a towel soaked in water and a low concentration of ethanol to avoid odor interfering with the results of the next rat).

### Sucrose preference test

Females were first acclimatized by placing two water bottles containing 1% sucrose water in each cage for 24 h and allowed to drink ad libitum. After 24 h fasting from food and water for 1 day, two weighed bottles of water (one bottle of 1% sucrose water and one bottle of pure water) were given to each female rat at 10:00 h the following day. After drinking freely for 1 h, the positions of the two bottles were changed to avoid the habitual drinking behavior affecting the experimental results. Measurements were taken after a further 1 h. Testing was carried out on day 21 of stress. Measurements include: i) Sucrose water consumption; ii) Pure water consumption; iii) Total liquid consumption; and iv) 1% sucrose preference percentage = sucrose water consumption/pure water consumption × 100%.

### Divide the offspring

The day the pups are born is considered day 0 (PND 0), and the male and female can be distinguished by the distance from the genitalia to the anal line. Check pup weights, sex ratios, and litter sizes and keep male offspring for further experiments on postnatal day 1 (PND 1). There were a total of 56 male neonates in the PC group and a total of 53 male neonates in the PS group. Birth weights of neonates were measured at PND 1, as mothers are susceptible to environmental stimuli after delivery (23). To avoid differentiation caused by differences in the number of male offspring reared between each litter, females were fed six male offspring in each litter. Some male offspring (*n* = 7–8) were randomly brain sampled at PND 0; all experimental samples were samples of male offspring from different litters. Other male offspring from the PC and PS groups received regular feeding until day 60 when the males were executed as adults.

### Sample collection

All animals were under isoflurane anesthesia. Under deep anesthesia, the animals were euthanized with sodium pentobarbital (100 mg/kg, intraperitoneal injection) to obtain serum, spermatozoa, and hypothalamic and testicular tissues. The Animal Ethics Committee of Hubei University of Traditional Chinese Medicine approved (ethical clearance number HUCMS00298243) all procedures and husbandry conditions used in the study.

### Real-time quantitative PCR (qPCR) detection of hypothalamic PND 0 and PND 60 KISS1 and GR gene expression in male offspring

50 mg of the hypothalamus tissues of male offspring were extracted with 1 mL of TRIzol, and an ultra-micro spectrophotometer determined the RNA concentration. The first strand cDNA synthesis kit was used to transcribe RNA into cDNA. Reaction conditions: reaction at 25°C for 5 min, reverse transcription at 52°C for 15 min, denaturation at 83°C for 5 min, and annealing at 4°C for 10 min. Totally, 4 μL of cDNA (5× dilution) was added to 0.2 μL of each of the upstream and downstream primers for real-time PCR. The parameters of the cycling program were pre-denaturing at 93°C for 10 min, denaturing at 95°C for 10 s, and annealing at 60°C for 30 seconds, for 40 cycles. Wuhan Seville Biotechnology Co provided the primers (sequences of primer sets are shown in Table 1). The amplification curve lysis curves were plotted, and the results were analyzed using the 2^-ΔΔCt^ calculation.

**Table 1:** Sequences of real-time PCR primer sets used in this study.

Gene	Genus	Primer sequences (5′–3′)	Length (bp)
GAPDH	Rat	F:CTGGAGAAACCTGCCAAGTATG	138
		R: TTCAGCTCTGGGATGACCTT	
GR	Rat	F:GCCGCTATCGGAAATGTCTT	204
		R: ACAACACCTCGGGTTCAATCAC	
KISS1	Rat	F:TGTCAGCCTACAACTGGAACTCC	147
		R: TTGCACAAGTCTAGAAGCTCCCT	

### Determination of GC and male offspring GC, GnRH, FSH, and AMH levels in female rats

Blood samples were collected from the heart on day 21 of stress in female rats and after deep anesthesia of sodium pentobarbital (100 mg/kg, peritoneal injection) injection of male offspring PND60. All blood samples were collected between 12:00 h and 12:10 h on the same day. Whole blood specimens collected in serum separator tubes were left at room temperature for 2 h or overnight at 4°C and then centrifuged at 1000 ***g*** for 20 min to separate the serum. Serum samples are stored at −20°C for 3 days until glucocorticoids, gonadotropin-releasing hormone, follicle growth hormone, and anti-müllerian hormone are measured by ELISA kits. All analyses were performed on the same day to avoid multiple freeze-thaw cycles. Because of the pulsatile nature of GnRH secretion, spot measurements may not be representative of the overall activity of the HPG axis.

### Assessment of testicular morphology and sperm quality parameters in male offspring of PND 60

Male offspring PND 60 right testicular tissue was fixed with testicular fixative, paraffin-embedded sections were stained with HE, and the extent of lesions and number of supporting cells in testicular sections were observed under a light microscope, and scores were tallied for each pathological change score. Male offspring were anesthetized with sodium pentobarbital (100 mg/kg, intraperitoneal injection), the epididymal cauda was removed, placed in pre-warmed phosphate buffer (PBS) at 37°C and incubated in a water bath at 37°C for 1 h. Sperm suspensions were prepared, filtered through a 200 mesh sieve, and sperm separated from tissue debris then analyzed for sperm density and motility.

### Immunofluorescence assay for the detection of FSH-R and CX43 protein expression in the testes of male offspring of PND 60

The embedded wax block was sliced at 4 μm, closed with 3% H_2_O_2_ solution for 10 min at room temperature, and rinsed with PBS and microwave antigen repair in 0.01% citrate buffer. After incubation at 37°C for 22 min, protected from light and closed, add 1:200 diluted FSH-R and CX43 antibodies overnight in the refrigerator at 4°C. The samples were removed, washed in PBS, incubated with rabbit anti-sheep secondary antibody, DAB color development, blocked, and photographed by fluorescence microscopy. The average fluorescence intensity of the samples was analyzed using Image Pro Plus 5 images.

### Western blot for male progeny PND 0 and PND 60 hypothalamic GR, KISS1-R, and PND 60 testis BCL-2, BAX, Caspase-3, FSH-R, CX43 protein levels

50 mg of fresh hypothalamic and testicular tissue was taken from male offspring. The protein was extracted from RIPA (radioimmunoprecipitation assay) lysate, and protein concentration was determined by the bicinchoninic acid (BCA) method. The protein samples were sampled, transferred, and blocked with β-actin, GR, KISS1-R, BCL-2, BAX, caspase-3, FSH-R, and CX43 primary antibodies (1:1000) and incubated overnight at 4°C in a refrigerator. After washing again, incubation with rabbit multiple antibody secondary antibody (1:1000), and membrane washing, the bands were developed with ECL ultrasensitive luminescent solution, imager camera, measurement and calculation of the integrated optical density; the ratio of the bands to the grey value of each group of internal reference β-actin was used as the relative expression of BCL-2, BAX, caspase-3, FSH-R, and CX43 proteins.

### Statistics analysis

Images were analyzed using ImageJ 7.0 image software, and data were analyzed using SPSS 22.0 software. Data were analyzed by *t*-test if normally distributed (Student’s *t*); if data did not conform to normal distribution, rank-sum test was used and results were expressed as median (25%, 75%). Statistical results *P* < 0.05 indicated statistical significance.

## Results

### Modeling prenatal stress through CUMS

**Figure 2:**
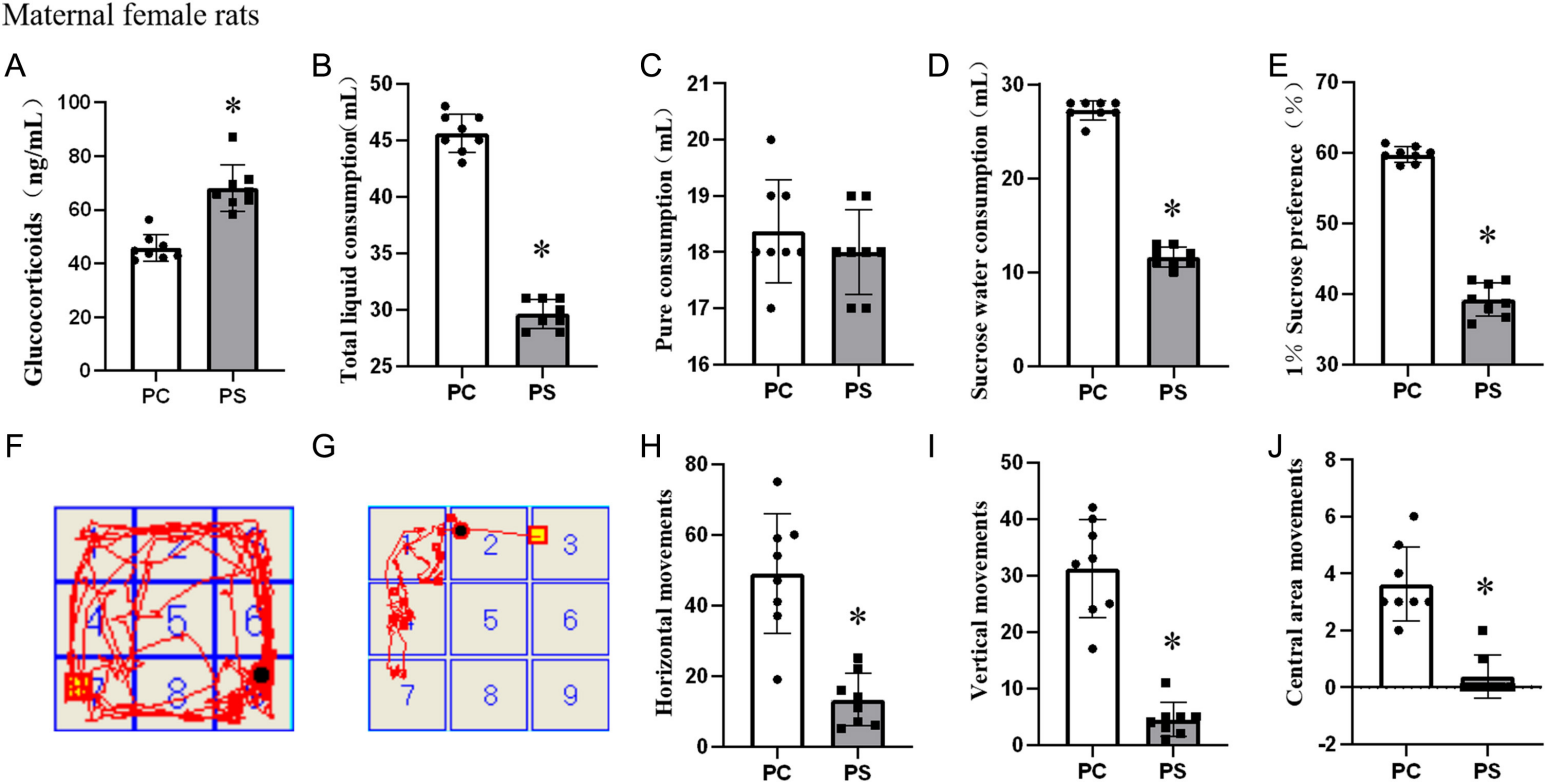
CUMS modeling of prenatal stress. (M(p25, p75)); *n* = 8; PC group, prenatal control group; prenatal control (PS) group, prenatal stress group. (A) Glucocorticoid levels in female rats of the maternal generation after CUMS modeling, *P < 0.05 (vs PS group, Wilcoxon rank-sum test). (B) Total fluid consumption of female rats in the maternal generation. (C) Pure water consumption in female rats of the maternal generation. (D) Sugar water consumption in female rats of the maternal generation. (E) Percentage of 1% sucrose preference in female offspring rats. (F) Action trajectories of female rats of the PC group in the open field test. (G) Action trajectories of female rats of the PS group in the open field test. (H) Horizontal locomotion scores in the minefield trials of female rat offspring. (I) Vertical locomotion scores in the minefield test of female rats of the maternal generation. (J) Number of times crossing the center in the minefield test of female rats of the maternal generation. PC group.

A prenatal stress model was established by CUMS (Fig. 2), and the glucocorticoid levels in the PS group were significantly higher (*P* < 0.05) than those in the PC group after CUMS modeling in the maternally-generated female rats, as shown in Fig. 2A. Figure 2B shows the total fluid consumption of female rats in the maternal generation; the level of total fluid consumption was significantly lower in the PS group compared with the PC group (*P* < 0.05). There was no statistically significant difference in pure water consumption between the two groups (Fig. 2C). Figure 2D shows that the level of sugar and water consumption was significantly decreased (*P* < 0.05) in the PS group compared to the PC group. In 1% sucrose preference percentage (Fig. 2E), the PS group was statistically significantly lower than the PC group (*P* < 0.05). Figure 2F and G show the action trajectories of PC and PS groups in the mine experiment, respectively. Figure 2H, I and J show the horizontal locomotion score, vertical locomotion score, and the number of entries into the central zone of female rats in the minefield experiment, respectively. Compared with the PC group, the PS horizontal locomotion score was significantly reduced (*P* < 0.05) (Fig. 2H). The PS vertical locomotion score was significantly lower (*P* < 0.05) than that of the PC group (Fig. 2I). The number of times of entering the central area in the PS group was significantly lower (*P* < 0.05) than that of the PC group (Fig. 2J).

### Effect of prenatal stress on litter size, sex ratio, and birth weight of male offspring

The total number of pups born was 109 in both groups, with a total of 56 male pups in the PC group and 53 male pups in the PS group. The male pup rate and birth weight were significantly lower (*P* < 0.05) in the PS group compared to the PC group (Table 2).

**Table 2:** Effect of prenatal stress on litter size, sex ratio, and birth weight of male offspring. Data are represented as mean ± s.e.m. from PC or PS male progeny.

Offspring	PC	PS
Total offspring number	109	109
Total Male rats	56	53
Birth weight of male rats (g)	6.92 ± 0.58	5.91 ± 0.72^#^

^#^*P* < 0.05 (vs pc group, Student’s *t*-test).

### Effects of prenatal stress on the KISS1 system in male offspring pups (PND 0) and adults (PND 60)

**Figure 3:**
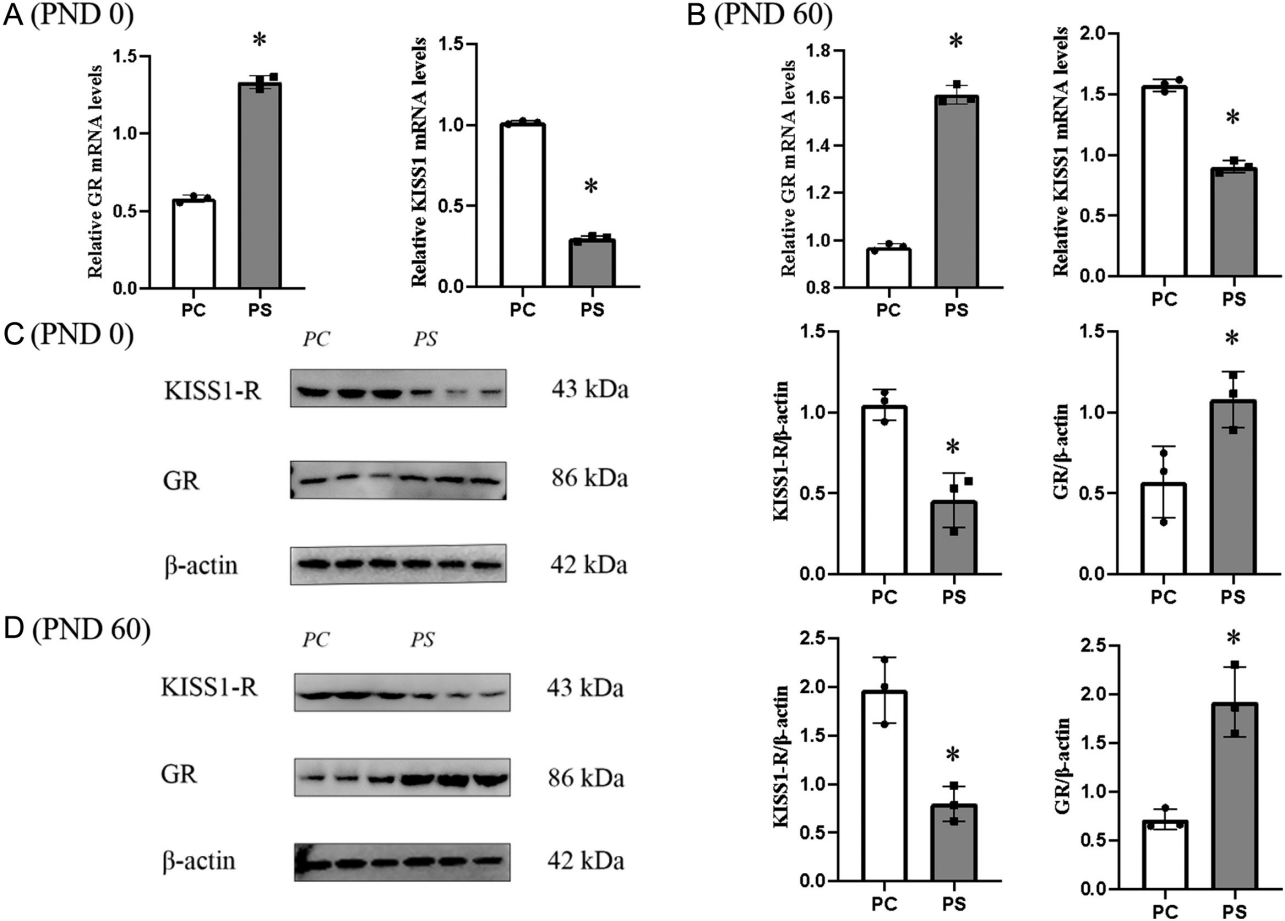
Effects of prenatal stress on the KISS1 system in male offspring pups (PND 0) and adults (PND 60). PC group, male offspring of the prenatal control group; PS group, male offspring of the prenatal stress group. *P < 0.05 (vs PC group, Student’s *t*-test), *n* = 3. (A) Relative expression of hypothalamic KISS1, GR genes in male offspring pups (PND 0). (B) Relative expression of KISS1, GR genes in the hypothalamus of adult male progeny (PND 60). (C) Relative expression of KISS1-R, GR proteins in the hypothalamus of male pups (PND 0). (D) Relative expression of KISS-R and GR proteins in the hypothalamus of adult male progeny (PND 60).

The relative expression of GR and KISS1-R proteins as well as the expression of GR and KISS1 genes were evaluated in pups and adults to determine the effects of prenatal stress on the KISS1 system in pups and adults (Fig. 3). During the pup period, the KISS1 mRNA level was significantly lower (*P* < 0.05) in the PS group compared to the PC group (Fig. 3A), and the GR mRNA level was significantly higher (*P* < 0.05) in the PS group compared to the PC group (Fig. 3A); the relative expression of the KISS-R protein was significantly lower (*P* < 0.05) in the PS group compared to the PC group (Fig. 3C), and the relative expression of the GR protein was was significantly higher (*P* < 0.05) than that of the PC group (Fig. 3C). In adulthood, the KISS1 mRNA level was significantly lower (*P* < 0.05) in the PS group compared with the PC group (Fig. 3B), and the GR mRNA level in the PS group was significantly higher (*P* < 0.05) than that in the PC group (Fig. 3B); the relative expression of the KISS-R protein in the PS group compared with that in the PC group was significantly lower (*P* < 0.05) (Fig. 3D), and the relative expression of the GR protein in the PS group was significantly higher (*P* < 0.05) than that of PC group (Fig. 3D).

### Effects of prenatal stress on spermatogenic function in adult (PND 60) male offspring

**Figure 4:**
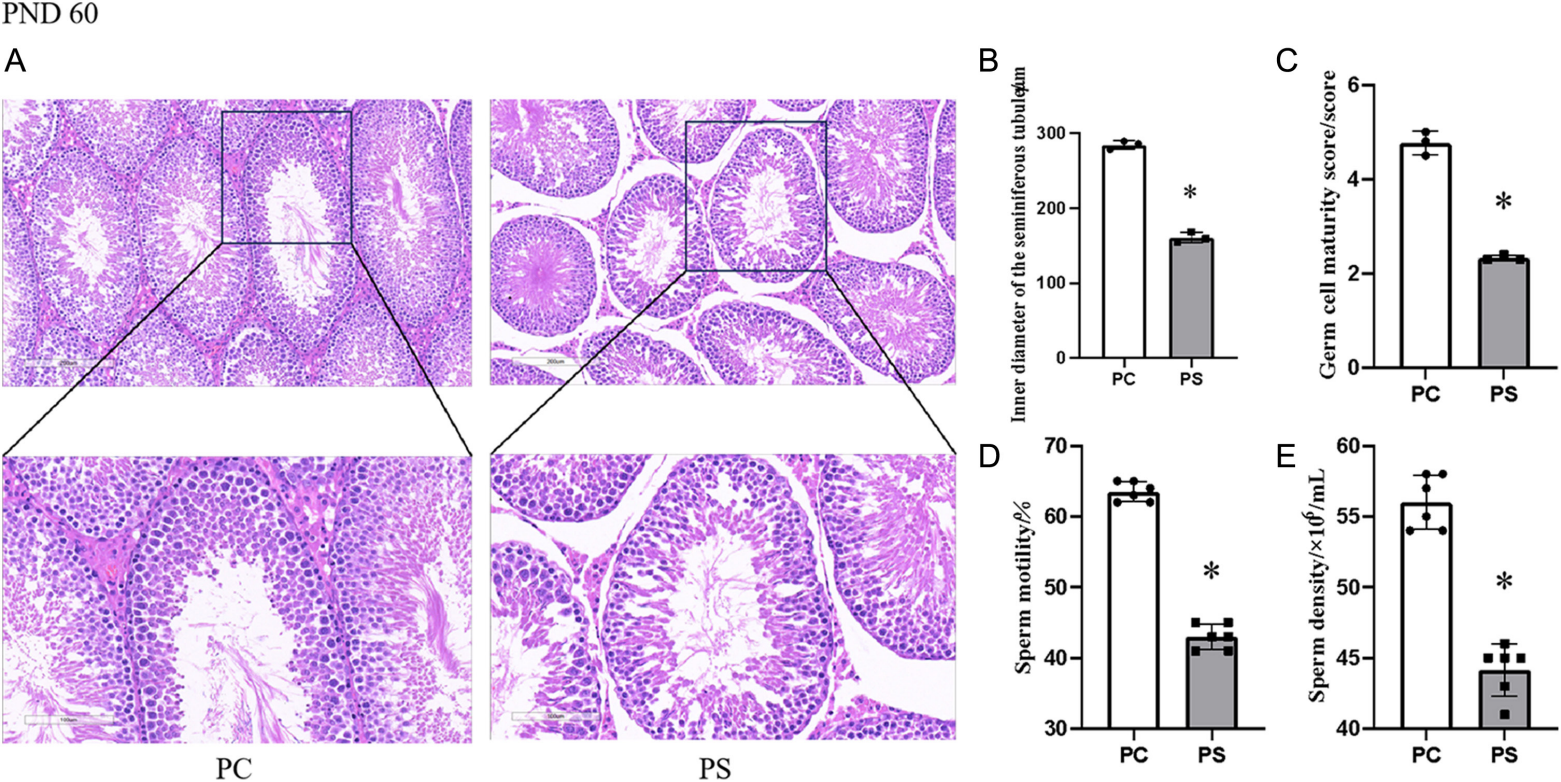
Effect of prenatal stress on spermatogenic function in adult (PND 60) male offspring. PC group, male offspring of the prenatal control group; PS group, male offspring of the prenatal stress group. (A) HE staining of testicular sections from the PC and PS groups. (B) Internal diameter of testicular seminiferous tubules in adult male offspring, *P < 0.05 (vs PC group, Student’s *t*-test). *n* = 3. (C) Germ cell maturity score of adult male offspring. (D) Sperm motility of adult male offspring, *P < 0.05 (vs PC group, Student’s *t*-test), *n* = 6. (E) Sperm density in adult male offspring.

Prenatal stress led to a decline in reproductive function in male offspring in adulthood (Fig. 4). The PC group had intact testicular tissue structure, dense structure of the seminiferous tubules, uniform distribution, smooth and uniform membranes, regular arrangement of the supporting cells and spermatogonial cells at all levels, and a large number of mature spermatozoa were present in the center of the lumen (Fig. 4A); in the PS group, some of the seminiferous tubules were shrunken, the membranes were thickened, and there were fewer germ cell layers at all levels, with a larger number of mature spermatozoa within the lumen (Fig. 4B). Fewer (Fig. 4B); statistical scoring results showed that compared with the PC group, the internal diameter of testicular seminiferous tubules and the scores of the degree of germ cell maturation in the PS group were significantly lower (*P* < 0.05) (Fig. 4C, D). Figure 4E and F show the sperm quality assessment in adulthood, and the levels of sperm density and motility were significantly lower in the PS group compared with the PC group (*P* < 0.05).

### Adult (PND 60) male progeny FSH-R/CX43 induces apoptosis in supporting cells

**Figure 5:**
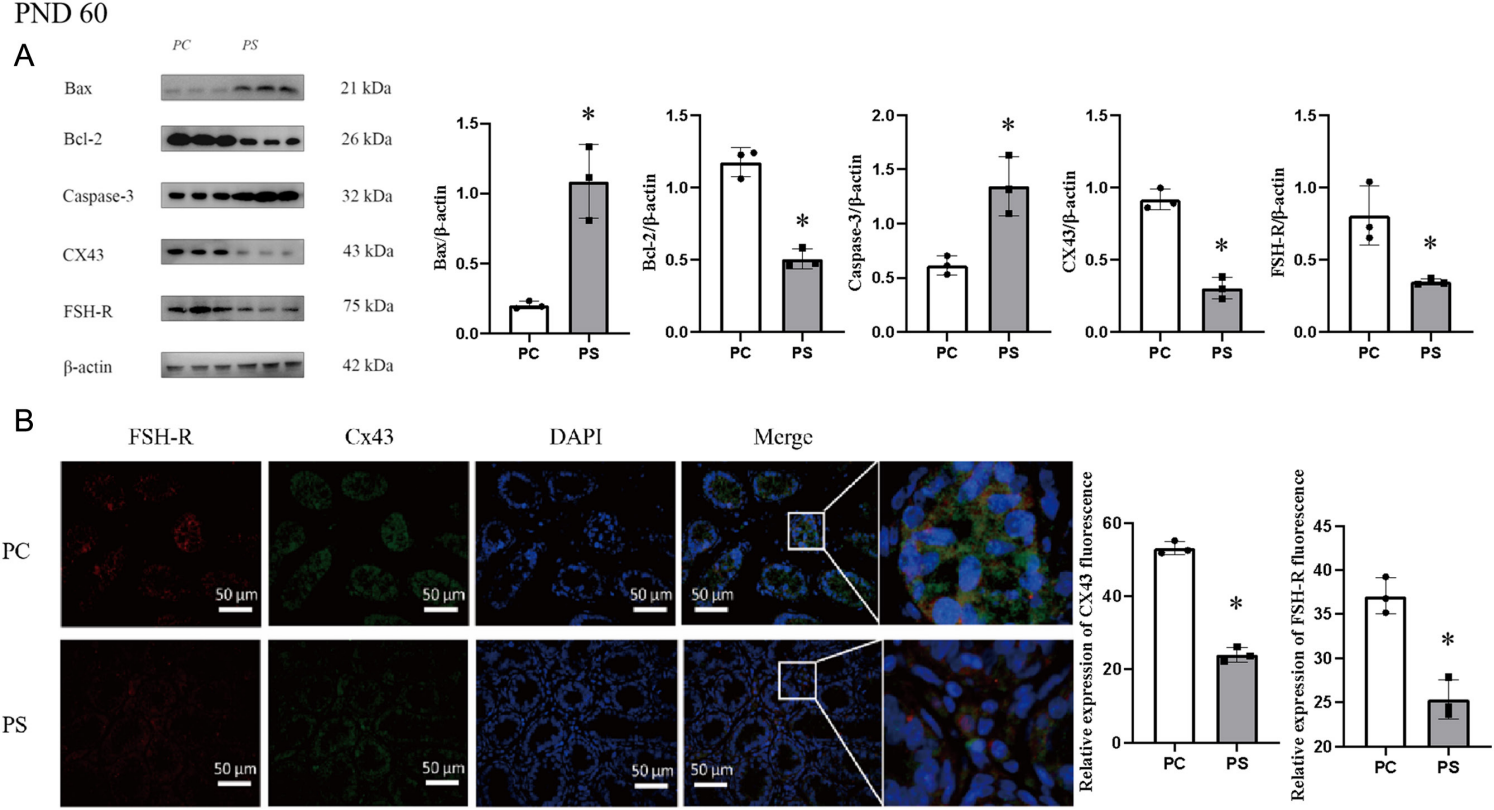
Adult male progeny FSH-R/CX43 induced supported apoptosis. PC group, male offspring of the prenatal control group; PS group, male offspring of the prenatal stress group. *P < 0.05 (vs PC group, Student’s *t*-test), *n* = 3. (A) Relative expression of Bax, Bcl-2, Caspase-3, CX43 and FSH-R proteins in the testis of adult male progeny. (B) Relative fluorescent expression of FSH-R, CX43 in the testes of adult male offspring.

In adulthood, the relative expression of Bcl-2, CX43, and FSH-R proteins was significantly lower (*P* < 0.05) in the PS group compared with the PC group (Fig. 5A), and the relative expression of Bax and Caspase-3 proteins in the PS group was significantly higher than that in the PC group (*P* < 0.05) (Fig. 5A). Figure 5B shows the immunofluorescence staining of CX43 and FSH-R proteins in pre-adult testicular tissues, and the relative fluorescence expression of CX43 and FSH-R was significantly lower in the PS group compared with the PC group (*P* < 0.05).

### Effects of prenatal stress on hormones in adult (PND 60) male offspring

**Figure 6:**
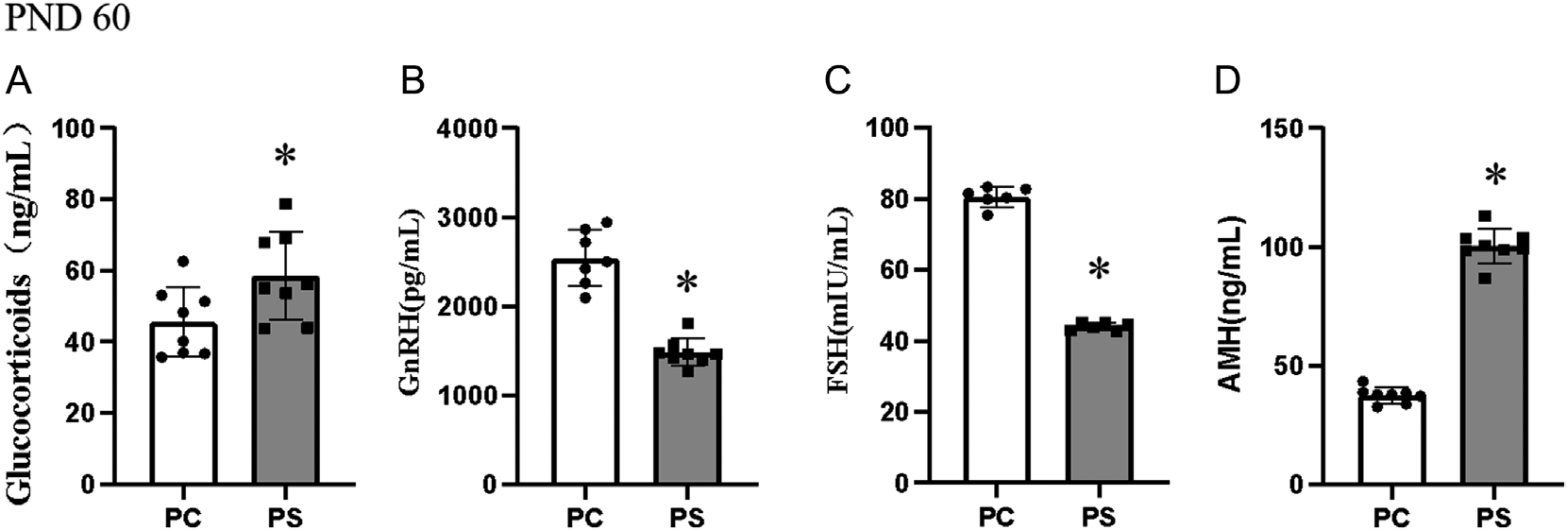
Differences in hormone levels in adult (PND 60) male offspring. PC group, male offspring of the prenatal control group; PS group, male offspring of the prenatal stress group. *P < 0.05 (vs PC group, Student’s *t*-test), *n* = 8. (A) Glucocorticoid levels in adult (PND 60) male offspring. (B) GnRH levels in adult (PND 60) male offspring. (C) FSH levels in adult (PND 60) male offspring. (D) AMH levels in adult (PND 60) male offspring.

We compared the differences in hormone levels in adult (PND 60) male offspring (Fig. 6), and the PS group of adult male offspring were still in the stress stage compared to the PC group, and the glucocorticoid levels were significantly higher in the PS group than in the PC group (*P* < 0.05), as shown in Fig. 6A. Figure 6B shows the GnRH secretion level of adult male offspring, which was significantly lower (*P* < 0.05) in the PS group compared with the PC group. Among the FSH secretion levels in both groups, the FSH secretion level in the PS group remained significantly lower than that in the PC group (*P* < 0.05) (Fig. 6C). Figure 6D shows the level of AMH secretion in male offspring in adulthood (PND 60), which was significantly higher in the PS group compared to the PC group (*P* < 0.05).

## Discussion

Early life adversity is associated with a distant risk of disease, and exposure to stressors during pregnancy can lead to increased susceptibility to disease in offspring (24). Among prenatal stress, glucocorticoids, the primary mediators of the stress response, increase placental permeability, leading to prenatal fetal glucocorticoid exposure and interference with fetal tissue and organ development (10, 11, 12, 13). Our study shows that prenatal stress inhibits the HPG axis by prenatally programming the hypothalamic KISS1 system in male offspring, affecting the maturation of supporting cells and altering the microenvironment of spermatogenesis, leading to reduced sperm quality. In addition, we found that antenatal stress can lead to low birth weight in newborns. However, birth weight is an important marker of fetal health, and low birth weight is associated with fetal mortality, growth retardation, and adult morbidity (25). Current studies collectively show that intrauterine glucocorticoid exposure due to antenatal stress is strongly associated with low birth weight (26, 27, 28) and that prolonged exposure to high levels of glucocorticoids leads to increased vascular resistance in the fetal placental body circulation (29), inducing intrauterine growth restriction (IUGR) (30).

Prenatal stress-induced changes in reproductive function in male offspring may be due to prenatal programming of the hypothalamic KISS1 system, which has a stimulatory role in pulsatile GnRH/LH secretion (31). Kisspeptin is mainly expressed in KISS1 neurons located in the arcuate nucleus of the hypothalamus (ARC) and the anterior ventral periventricular nucleus (AVPV) region (32). Studies have shown that upregulation of strongorphin expression within AVPV kisspeptin neurons following stress-induced increases in glucocorticoid levels leads to reduced kisspeptin secretion (33). In addition, the glucocorticoid receptor (GR) is expressed in mouse hypothalamic kisspeptin neuronal cells (34), and dexamethasone inhibits the transcriptional expression of Kiss1 mRNA and the expression level of Kisspeptin protein in hypothalamic GT1-7 neuronal cells. In contrast, the glucocorticoid receptor blocker RU486 antagonizes this effect (35). These findings suggest that GR on hypothalamic kisspeptin neurons mediates prenatal stress-induced negative changes in the KISS1 system. Thus, prenatal stress may lead to prenatal programming of the hypothalamic KISS1 system in newborns, leading to altered reproductive function during growth by inhibiting the HPG axis in male offspring. The current male offspring of PS show increased GR gene expression at birth and still have higher glucocorticoid and GR levels than controls in adulthood. This suggests that the male offspring remain stressed, which may also accelerate negative changes in the hypothalamic KISS system.

Our study found that prenatal stress decreased hypothalamic KISS1 gene expression at birth, and the subsequent trend in expression continued to decrease in adulthood. Similarly, PS significantly suppressed hypothalamic GPR54 receptor expression at birth versus adulthood, suggesting that prenatal stress causes developmental programming of the hypothalamic KISS1 system and that its gene expression trends do not change with life stage. Along with alterations in the hypothalamic KISS1 system, their levels of GnRH secretion during adulthood were significantly lower than those of the control group. At the same time, we observed that their FSH and FSH-R expressions were also at low levels. FSH is a glycoprotein consisting of alpha and beta subunits, whereas FSHR is only expressed in the cell membrane of supporting cells (36). FSH signaling is elevated in adolescence to stimulate Sertoli cell proliferation and in adulthood to drive the production of regulatory molecules and nutrients required for spermatogenesis. FSH circulating levels correlate with the adult Sertoli cell numbers (37). In men, low fertility with a quantitative reduction in spermatogenesis occurs without FSHR function (38), while mutations in the FSHβ subunit can lead to azoospermia and infertility (39). At the same time, in the histomorphology of the testis, we observed a reduced number of supporting cells in the PS testis, a finding that is consistent with previous findings. To further assess the maturity of Sertoli cells, we measured the expression of adult anti-mullerian hormone (AMH). AMH is a glycoprotein hormone produced mainly by testicular supporting cells (40). AMH is a unique biomarker for immature Sertoli cells, which progressively express less AMH during pubertal development (41). It was shown that AMH at high concentrations significantly increased the expression of Caspase-3 and Bax proteins and decreased the expression of Bcl-2 protein in Sertoli cells, demonstrating that apoptosis was promoted at high concentrations of AMH (42). These studies are also supported by our results, which show that AMH is elevated in PS male offspring with increased expression of Caspase-3 and Bax proteins and decreased expression of Bcl-2 protein. Spermatogenesis is a complex process, and the microenvironment in which spermatogenesis occurs is equally crucial. Sertoli cells promote spermatogenesis through paracrine action to provide the necessary nutrients and factors, and memorable connections between Sertoli cells near the basement membrane produce the blood-testis barrier (BTB). BTB consists of coexisting tight junctions (TJ), basal ectoplasmic specialization (ES), and bridging granule-like junctions (43). Connexin 43 (Cx43) is a gap-linked integral membrane protein that cycles throughout the germinal epithelial cycle of spermatogenesis and is essential for TJ recombination in BTB (44). We, therefore, assessed BTB by testing CX43, and our results found a similar reduction in CX43 expression in the offspring of PS males, suggesting that the spermatogenic microenvironment is also altered in the offspring of PS males. Finally, our sperm test in the offspring of PS men showed a decrease in total sperm count and sperm motility and a higher degree of sperm apoptosis than in the control group.

This study found that prenatal stress affects the programming of the hypothalamic KISS1 system in male offspring, leading to long-term effects on the hypothalamic testicular axis and later affecting reproductive function. In addition, antenatal stress leads to elevated glucocorticoids, causing intrauterine growth retardation through intrauterine glucocorticoid exposure. In the context of preventive medicine, this study provides evidence that maternal stress management is critical to the reproductive function of offspring and will contribute to the treatment of reproductive disorders in patients with intrauterine growth retardation.

## Declaration of interest

The authors declare that they have no financial and personal relationships with other people or organizations that can inappropriately influence their work, and there is no professional or other personal interest of any nature or kind in any product, service, or company.

## Funding

National Natural Science Foundation of Chinahttp://dx.doi.org/10.13039/501100001809 ( no. 82104704 ); 2021 Hubei Universityhttp://dx.doi.org/10.13039/501100017589 of Traditional Chinese Medicine Research Team Building Program (TCM (2021) 183 )
